# Identification of immune-based prostate cancer subtypes using mRNA expression

**DOI:** 10.1042/BSR20201533

**Published:** 2021-01-04

**Authors:** Jukun Song, Wei Wang, Yiwen Yuan, Yong Ban, Jiaming Su, Dongbo Yuan, Weihong Chen, Jianguo Zhu

**Affiliations:** 1 School of Medicine, Guizhou University, Guizhou, China; 2 Department of Oral and Maxillofacial Surgery, Guizhou Provincial People’s Hospital, Guizhou, China; 3 Department of Urology, Guizhou Provincial People’s Hospital, Guizhou, China; 4 Department of Clinical Medicine, Guizhou Medical University, Guizhou, China

**Keywords:** CIBERSORT algorithm, Clinical application, Immunotherapy, Prostate cancer, Tumor-infiltrating immune cells

## Abstract

Immune infiltration in Prostate Cancer (PCa) was reported to be strongly associated with clinical outcomes. However, previous research could not elucidate the diversity of different immune cell types that contribute to the functioning of the immune response system. In the present study, the CIBERSORT method was employed to evaluate the relative proportions of immune cell profiling in PCa samples, adjacent tumor samples and normal samples. Three types of molecular classification were identified in tumor samples using the ‘CancerSubtypes’ package of the R software. Each subtype had specific molecular and clinical characteristics. In addition, functional enrichment was analyzed in each subtype. The submap and Tumor Immune Dysfunction and Exclusion (TIDE) algorithms were also used to predict clinical response to the immune checkpoint blockade. Moreover, the Genomics of Drug Sensitivity in Cancer (GDSC) database was employed to screen for potential chemotherapeutic targets for the treatment of PCa. The results showed that Cluster I was associated with advanced PCa and was more likely to respond to immunotherapy. The findings demonstrated that differences in immune responses may be important drivers of PCa progression and response to treatment. Therefore, this comprehensive assessment of the 22 immune cell types in the PCa Tumor Environment (TEM) provides insights on the mechanisms of tumor response to immunotherapy and may help clinicians explore the development of new drugs.

## Background

Prostate Cancer (PCa) is the most common malignancy in Europe and the United States. The disease has resulted in the second highest number of mortalities, after breast cancer, in American male patients [1]. Additionally, the American Cancer Society reported 174650 new PCa cases in 2019 alone ranking first and accounting for 20% of all new male cancer cases. Moreover, 31620 deaths were reported from PCa in 2019, accounting for 10% of all cancer-related deaths [2]. PCa is the leading major type of tumor in 28 European countries and the second most prominent type in 7 other countries [3]. Furthermore, there are obvious ethnic differences in the incidence of PCa. For instance, the incidence and mortality rate of PCa in China is lower than that of Western countries including Europe and the United States. However, given the recent changes in society and people’s lifestyles, PCa has become common among males with increased incidence rates every year [4]. In addition, PCa is a heterogeneous disease that can vary greatly within the same tumor [5]. The significant differences in incidence and morbidity may be due to genomic instability and changes associated with various PCa risk factors.

Early treatment of PCa through androgen deprivation therapy was shown to be effective although ultimate development of the disease in a hormone-independent fashion presents a challenge [6]. Additionally, the cancer phenotype is not only defined by the intrinsic activity of tumor cells but also by immune cells recruited and attracted in the tumor microenvironment. Presently, the role of immune cells in the tumor microenvironment during the development of cancer, particularly in PCa, remains largely unclear.

A growing body of evidence demonstrated that Tumor Infiltrating Leukocytes (TILs), including B cells, T cells, dendritic cells, macrophages, neutrophils, monocytes and polar cells, might control malignant growth. In addition, TILs are important components of the tumor microenvironment and can alter the immune status of cancers. The impact of TILs on the progression of tumors has been extensively documented [7–10]. In the present study, a newly developed deconvolution algorithm, CIBERSORT, was used to define leukocyte signature matrix (LM22) subsets of immune responses to examine their correlation with molecular subsets as well clinical features. The findings revealed distinct immune phenotypes for molecular PCa subclasses. In addition, the present investigation gives novel insights on possible immunotherapy against PCa.

## Materials and methods

### Gene expression datasets

Publicly available gene expression profiles were obtained from The Cancer Genome Atlas (TCGA) and GTEX databases. Healthy human prostate tissues were available from the GTEx (https://gtexportal.org/home/) database, while tumor and adjacent tumor samples samples were obtained from TCGA (https://cancergenome.nih.gov/). Additionally, RNA-seq profiles (FPKM values) and phenotype data were downloaded from the TCGA website. First, the voom (variance modeling at the observational level) method was used to convert the mRNA sequence data into matching results from the microarray data [11]. The gene IDs were then annotated and mRNA sequencing results normalized using the ‘limma’ package of the R software to average the repeated gene data and remove unavailable data [12]. The expression matrix and clinical characteristics of each patient were manually organized. Moreover, patients with full clinical pathology data and survival time of more than 30 days were included in the study.

### Inference of TILs using the CIBERSOFT algorithm

The CIBERSOFT algorithm is an analytical tool which accurately infers the relative levels of human immune cell types within a complex gene expression profile. The detailed description of LM22 is shown in the Supplementary Table S1. In the present study, the algorithm used the properties of 547 marker genes to characterize and quantify the relative scores for each immune cell subtype. In addition, it used Monte Carlo sampling to derive the deconvoluted *P*-value for each sample and this ensured robustness in the results. The standardized and processed gene expression dataset was uploaded to the CIBERSOFT website (https://cibersort.stanford.edu/index.php), which ran while utilizing 1000 aligned default signature matrices [13]. After using the CIBERSORT program, the distribution of the LM22 subtypes of TILs together with the results of the correlation coefficients, *P*-value and root mean square error (RMSE) could be used to assess the accuracy of the results in each sample. A *P*-value of ≤0.05 reflected the statistical significance of the deconvolution results per sample on all subsets of cells and was useful for screening outcomes with lower precision. Finally, 64 normal samples, 32 adjacent tumor samples and 351 tumor samples were selected for further analysis with a cut-off *P*-value of less than 0.05.

The study considered total T-cell fraction as the sum of CD8^+^ T cells, naive CD4^+^ T cells, resting and activated memory CD4^+^ T cells, follicular helper T cells, regulatory T cells (Tregs) and T cells γδ. In addition, total macrophage cell fraction was calculated as the sum of M0, M1 and M2 macrophage proportions. Finally, total B-cell fraction was considered as a sum of memory and naive B cells.

### Identification of prognostic LM22 immune cell subsets

The prognostic LM22 immune cell phenotypes were linked with Progression-Free Survival (PFS). First, the univariate Cox analysis and Kaplan–Meier survival analysis were performed to screen for prognostic immune cell types. Afterward, the multivariate Cox regression analysis was used to further validate 22 human immune cell phenotypes as prognostic factors.

### Unsupervised clustering analysis

A consensus cluster algorithm was applied to determine the number of clusters across the tumor samples. The NMF method in the ‘CancerSubtypes’ package of the R software was used to identify cancer subtypes from genomic data [14]. The performance of these clustering methods was evaluated using three common measures namely: (1) the log-rank test of Kaplan–Meier curves to evaluate the importance of survival differences between subtypes; (2) the average contour width (ASW) method of measuring cluster consistency to evaluate whether samples within a subtype or subtypes were more similar and (3) clustering heat maps to visualize sample clusters by separating color patches from each other.

### Screening of differentially expressed genes and differential immune cell types in each subclass

The limma package of the R software was used to identify Differentially Expressed Genes (DEGs) and specific immune cell types among 547 marker genes. This was done to uncover the potential subtype-specific immune-related genes and TIIC models in each cluster. The limma package used an empirical Bayesian approach to estimate changes in gene expression using moderated *t* tests. Finally, the DEGs and differential immune cell types were determined using significance criteria (adjusted *P*-value <0.05) [12].

### Pathways and biological functions differentially enriched in PCa subtypes

Functional enrichment analysis on DEGS was performed among PCa subtypes using the ‘Cluserprofiler’ package of the R software [15]. In addition, the Gene Ontology (GO) Biological Processes term and Kyoto Encyclopedia of Genes and Genomes (KEGG) terms were identified with a cutoff of *P*<0.05. Gene Set Enrichment Analysis (GSEA) was then conducted to unveil an overall pathway of the gene-set activity score for each sample [16]. The gene sets using the c2/c5 curated signatures were downloaded from the Molecular Signature Database (MSigDB) of the Broad Institute. Finally, significantly enriched pathways were identified based on the adjusted *P*-value <0.05.

### Mutation analysis

Mutation data in the MAF of 301 PCa patients were used for genetic and epigenetic analyses. The ‘maftools’ package of the R software was used to display the mutation profile of each subtype [17]. Moreover, maftools was used to determine the mutation rate of each gene and identify significant mutant genes in different subtypes (*P*<0.05).

### Prediction of chemo/immunotherapeutic response

The Tumor Immune Dysfunction and Exclusion (TIDE) algorithms [18] and subclass mapping [19] were used to predict clinical response to immune checkpoints. The chemotherapeutic response of each sample was also predicted based on the largest publicly available pharmacogenomics database [Pharmaceutical Sensitivity Genomics in Cancer (GDSC), https://www.cancerrxgene.org/] [20]. A total of 138 drugs showed potential for the treatment of cancer (Supplementary Table S2). Prediction was done using the ‘pRRophetic’ package of the R software. In the package, the half-maximal inhibitory concentration (IC_50_) of the samples was calculated through ridge regression and the prediction accuracy was assessed by ten-fold cross-validation based on the GDSC training set [21].

### Statistical analysis

All statistical tests were conducted through the R (version 3.5.2) software utilizing the Chi Square (χ^2^) or Fisher’s exact tests for categorical data accordingly. In addition, continuous data was analyzed using the Wilcoxon test (Mann–Whitney test) for two groups and the Kruskal–Wallis test for more than two groups [22]. The Kaplan–Meier curve [23] and Cox regression [24] were also used to screen for prognostic immune cell subclasses in the survival data. Survival analysis was performed using the ‘survival’ package of the R software. Fisher’s independent exact test was also used to statistically classify the relationship between clinical information and defined subtypes. A *P*-value of less than 0.05 was considered statistically significant in all the analyses.

## Results

### Inference of immune infiltration cells in the TME

**Figure 1:**
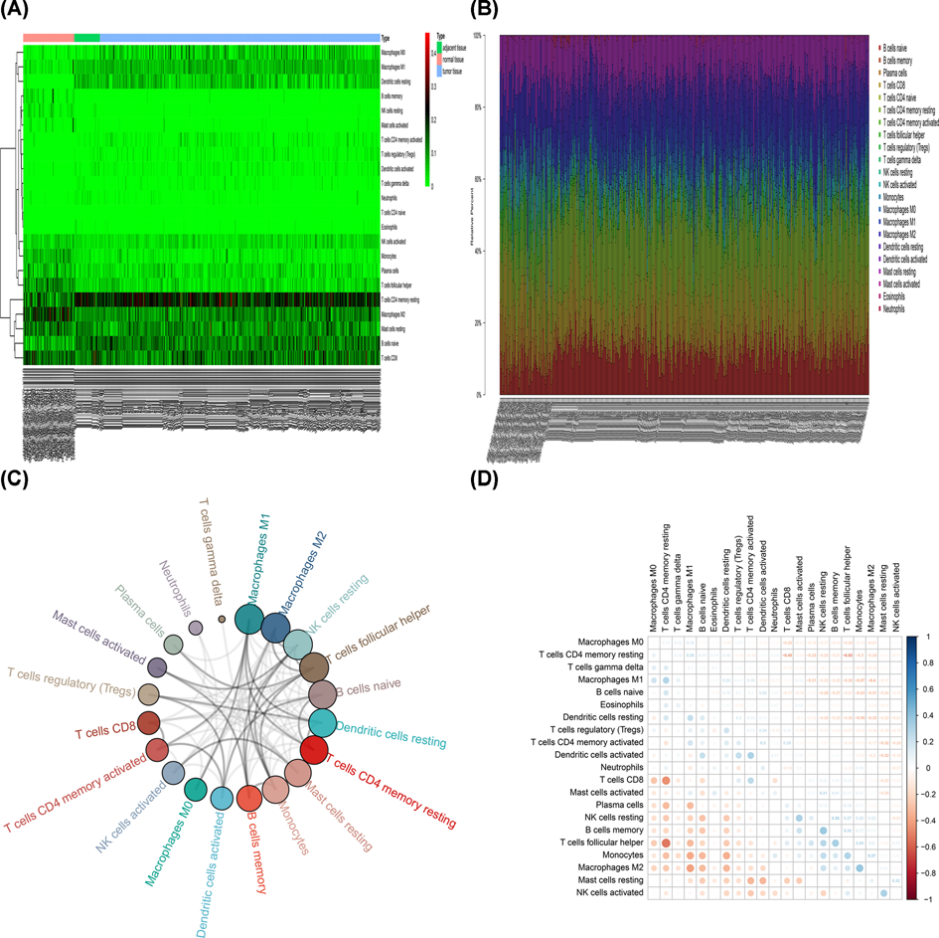
The performance of CIBERSOFT in estimating the composition of TILs in PCa, adjacent tumor and control tissues (**A**) A heat map. (**B**) Stacked histogram. (**C**) Circle graph. (**D**) The correlation matrix of 22 immune cell densities.

**Figure 2:**
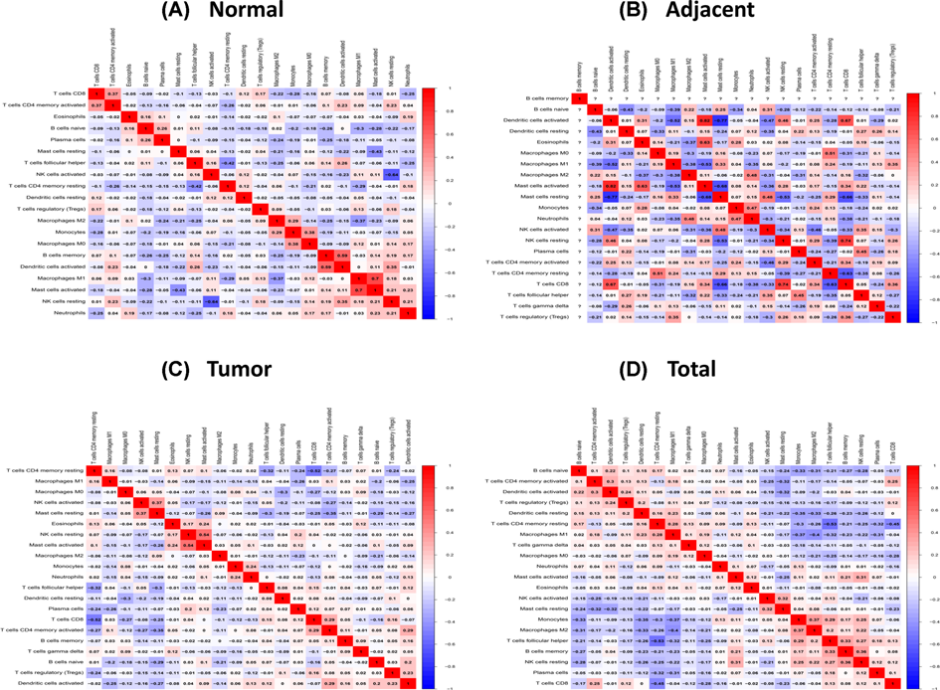
The correlation matrix of 22 immune proportions in the prostate tissues (**A**) The normal samples. (**B**) The adjacent tumor samples. (**C**) Tumor samples and (**D**) total samples.

The landscape of the cell infiltration models in the TME was inferred using the CIBERSOFT algorithm. Figure 1A,B summarizes the findings obtained from the 64 normal samples, 32 adjacent tumor samples and 351 tumor samples. Twenty-one types of TILs were detected in patients, while naive CD4^+^ T cells were absent from most of the samples, consistent with a previous report [25]. The Correlation matrix of all 22 immune cell densities in the TCGA cohort is shown in Figure 1C,D. In general, infiltration of LM22 differed among the samples. The highest proportion of immune cells was observed in normal samples, followed by adjacent tumor samples and finally tumor samples. Mutual interaction between immune cells in the normal and adjacent tumor samples was evident compared with the tumor samples (Figure 2A–D).

**Figure 3:**
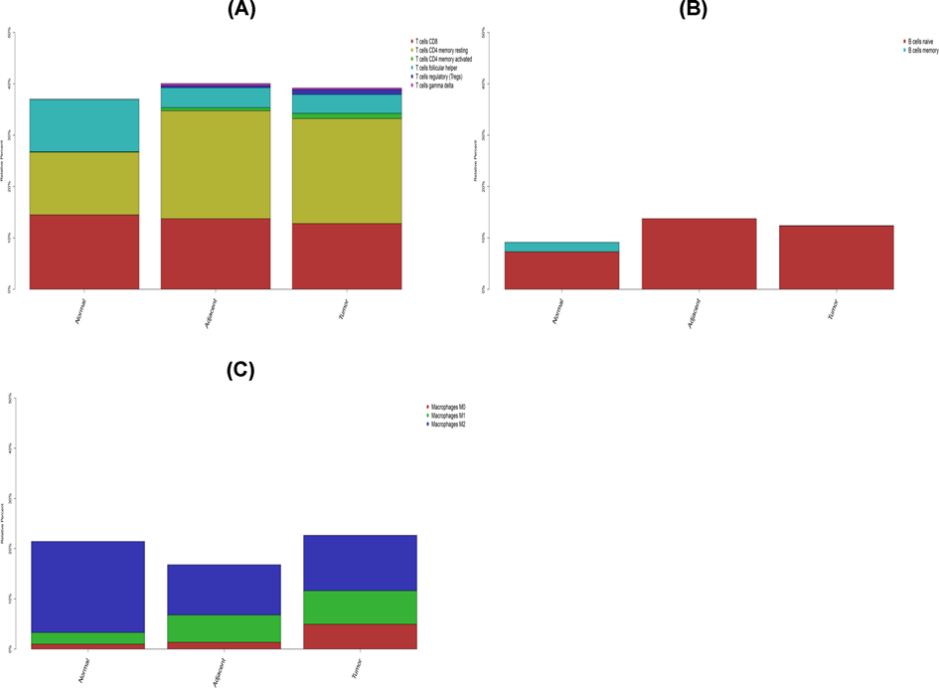
The stacked histogram shows the distribution of 22 immune cell infiltrations among normal tissues, adjacent tumor tissues and tumor tissues (**A**) Total T cells. (**B**) Total B cells. (**C**) Total macrophages.

Compared with normal tissues, the fraction of total T and B cells was higher in tumor and adjacent tumor tissues. Additionally, total macrophages were mainly observed in the tumor tissues (Figure 3A–C). Memory B cells were absent from both the tumor and adjacent tumor samples while macrophages M1 and M2 increased in the tumor samples. Moreover, T cells γδ and Tregs were found in both tumor and adjacent tumor samples. Therefore, the results suggested that the immune system was inhibited during tumor development.

**Figure 4:**
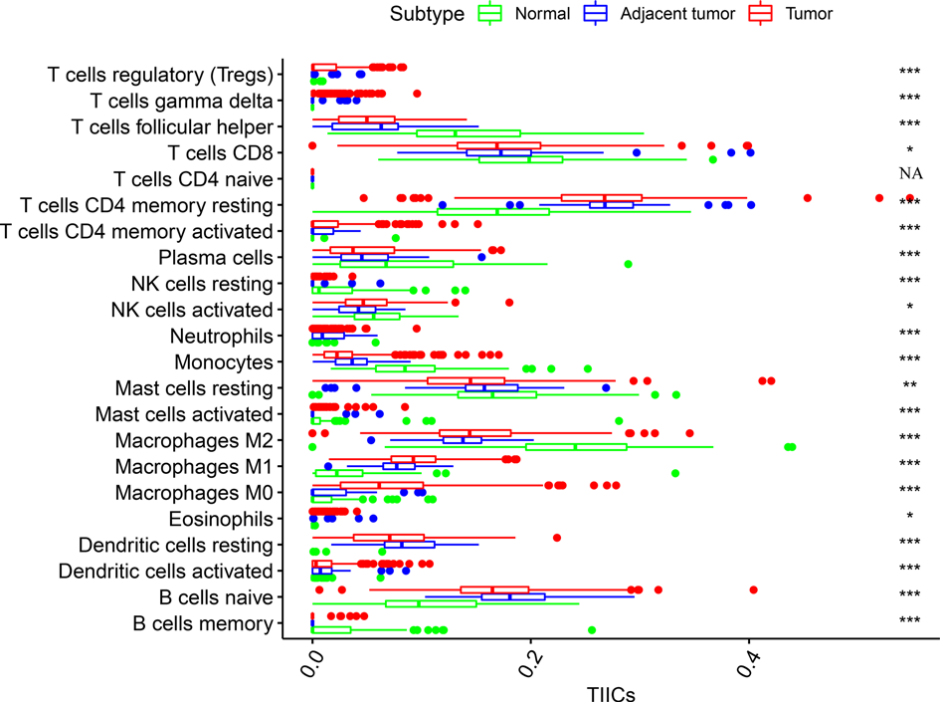
The box plot shows the difference between CIBERSOFT immune cell fractions and normal tissues, tumor and adjacent tumor tissues Kruskal–Wallis test was employed to test the difference among three groups, a *P*-value of less than 0.05 was considered statistically significant.

Additionally, the following cell types were enriched in the normal tissues; memory B cells, M2 macrophages, activated and resting mast cells, monocytes, resting and activated natural killer (NK) cells, CD8 T cells and follicular helper T cells. On the other hand, the following cell types were significantly present in the adjacent tumor tissues; naive B cells, activated and resting dendritic cells, M1 macrophages, activated and resting CD4 memory T cells and neutrophils. In the tumor tissues, naive B cells, activated and resting dendritic cells, M0 and M1 macrophages, activated and resting CD4 memory T cells and Tregs were mainly activated (Figure 4). Therefore, these findings demonstrated aberrant immune infiltration and its heterogeneity in PCa. This is a tightly regulated process that might play important roles in the development of tumor with significant clinical implications.

### Prognostic subsets of immune cells

**Figure 5:**
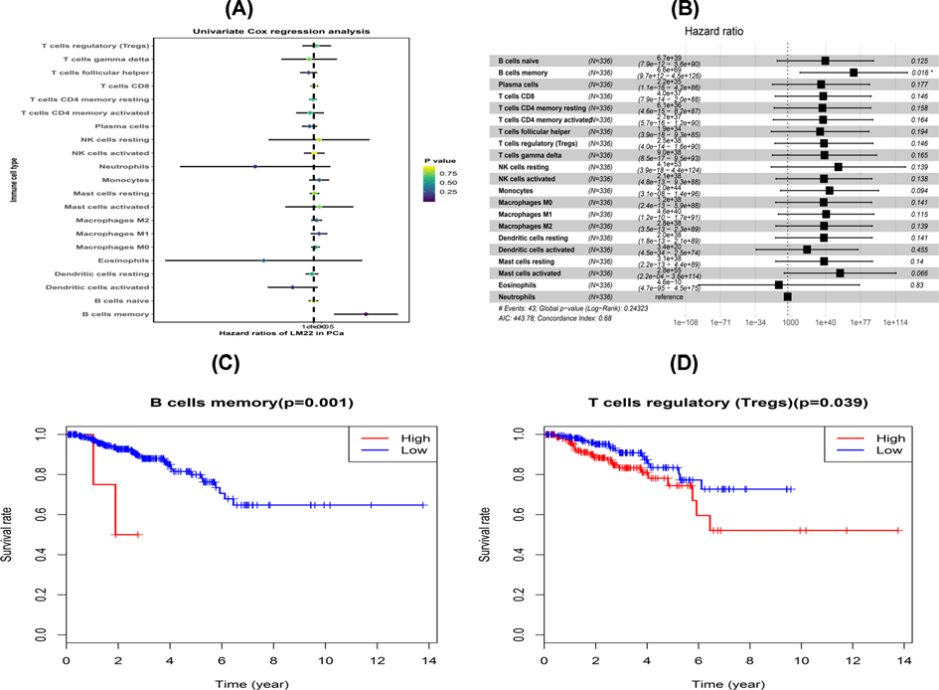
Identification of prognostic immune cells in the TCGA PCa cohort (**A**) The univariate Cox regression analysis and (**B**) multivariate Cox regression analysis. K–M survival analysis of two immune cells, including (**C**) memory B cells and (**D**) Tregs.

The univariate Cox regression analysis was performed to identify the prognostic subtypes of TILs in PCa. The results showed that memory B cells were significantly correlated with PFS with a cut-off *P*-value of less than 0.05 (Table 1 and Figure 5A). Similarly, multivariate Cox regression analysis revealed that memory B cells were closely associated with RFS (Table 2 and Figure 5B). Afterward, the Kaplan–Meier curve and log-rank tests were conducted on the immune cell subsets mentioned above and the results are shown in Figure 5C,D. The memory B cells and Tregs were significantly associated with RFS in PCa patients.

**Table 1:** The univariate Cox regression analysis was performed to identify the prognostic subtypes of TIICs in PCa

Immune cell	HR	HR.95L	HR.95H	*P*-value
B cells naive	0.663123	0.000441	996.5155	0.912357
B cells memory	2.98E+34	2.19E+13	4.06E+55	0.001388
Plasma cells	0.001334	1.20E-08	148.7424	0.264269
T cells CD8	2.075994	0.005774	746.3596	0.80779
T cells CD4 memory resting	0.253485	0.000796	80.75887	0.640723
T cells CD4 memory activated	0.003227	2.03E-12	5119968	0.595629
T cells follicular helper	0.000369	7.62E-10	178.984	0.236668
Tregs	79.00902	2.48E-08	2.52E+11	0.695531
T cells γδ	0.000811	3.25E-22	2.02E+15	0.741926
NK cells resting	2831.234	1.18E-30	6.77E+36	0.839372
NK cells activated	2.001437	2.76E-07	14491266	0.931388
Monocytes	3357.781	0.000786	1.43E+10	0.297283
Macrophages M0	11.70166	0.01414	9683.931	0.473023
Macrophages M1	2762.816	0.007955	9.6E+08	0.223474
Macrophages M2	70.26358	0.016501	299185.2	0.318604
Dendritic cells resting	0.056424	3.43E-06	927.6329	0.561617
Dendritic cells activated	1.11E-14	3.10E-31	399.1275	0.098531
Mast cells resting	2.98651	0.001924	4635.156	0.770392
Mast cells activated	4502.05	1.40E-19	1.45E+26	0.75037
Eosinophils	8.19E-34	5.81E-99	1.16E+32	0.319553
Neutrophils	2.02E-39	3.99E-89	1.02E+11	0.127062
T cells CD4 naive	NA	NA	NA	NA

**Table 2:** The multivariate Cox regression analysis was conducted to screen the prognostic subtypes of TIICs in PCa

Immune cell	HR	HR.95L	HR.95H	*P*-value
B cells naive	6.67E+39	7.90E-12	5.63E+90	0.125353
B cells memory	6.60E+69	9.74E+12	4.48E+126	0.016044
Plasma cells	2.19E+35	1.15E-16	4.18E+86	0.176776
T cells CD8	3.96E+37	7.90E-14	1.99E+88	0.146099
T cells CD4 memory resting	6.14E+36	4.59E-15	8.20E+87	0.158454
T cells CD4 memory activated	2.66E+37	5.70E-16	1.24E+90	0.163714
T cells follicular helper	1.92E+34	3.94E-18	9.33E+85	0.193608
Tregs	2.48E+38	3.96E-14	1.55E+90	0.146276
T cells γδ	8.99E+38	8.48E-17	9.52E+93	0.165286
NK cells resting	4.12E+53	3.86E-18	4.40E+124	0.139016
NK cells activated	2.11E+38	4.75E-13	9.35E+88	0.138054
Monocytes	2.04E+44	3.07E-08	1.36E+96	0.093777
Macrophages M0	1.19E+38	2.38E-13	5.92E+88	0.141039
Macrophages M1	4.64E+40	1.24E-10	1.74E+91	0.115014
Macrophages M2	2.82E+38	3.46E-13	2.29E+89	0.138805
Dendritic cells resting	1.98E+38	1.84E-13	2.13E+89	0.141324
Dendritic cells activated	3.37E+20	4.47E-34	2.54E+74	0.455198
Mast cells resting	3.10E+38	2.19E-13	4.37E+89	0.140241
Mast cells activated	2.80E+55	0.00022	3.57E+114	0.065967
Eosinophils	4.63E-10	4.73E-95	4.52E+75	0.82956
Neutrophils	NA	NA	NA	NA
T cells CD4 naive	NA	NA	NA	NA

### Patterns of immune cell infiltration in molecular PCa subclasses

**Figure 6:**
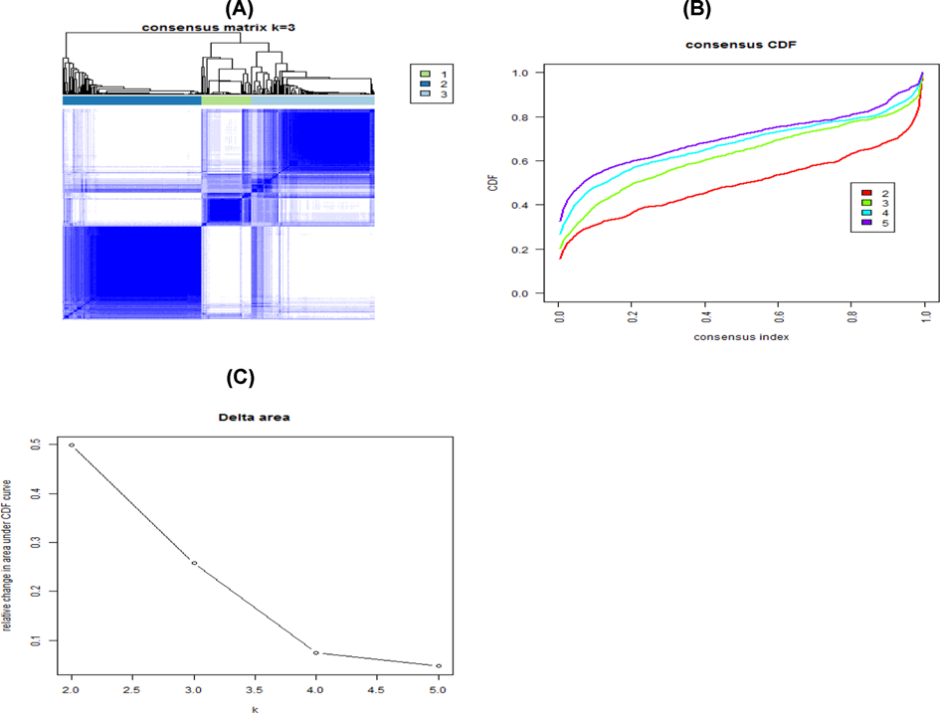
The cluster counts evaluated using unsupervised clustering analysis (**A**) The Consensus heat map. (**B**) The relative change in area under the CDF curve of K = 2–5. (**C**) CDF curve of K = 2–5.

**Figure 7:**
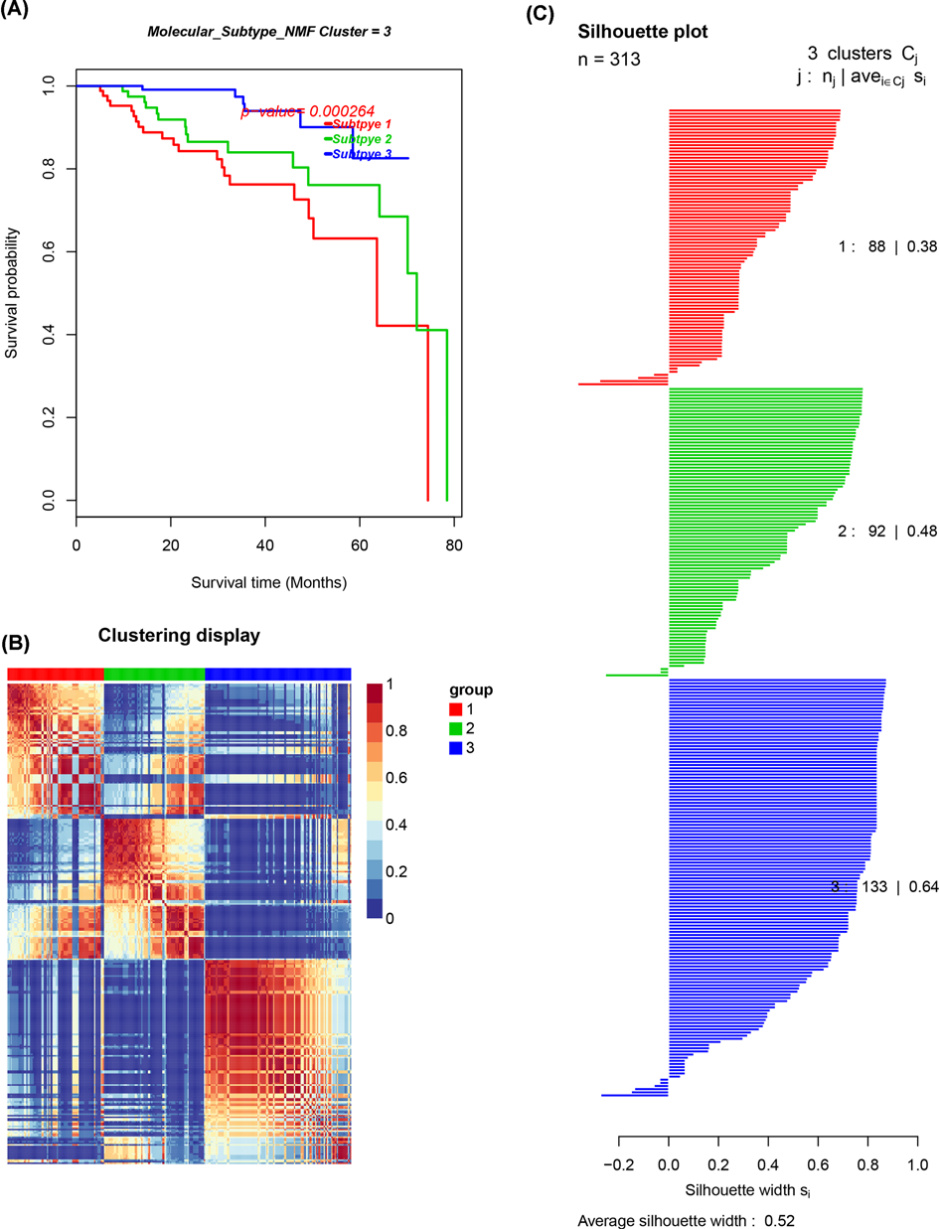
Identification of PCa subtypes using the NMF algorithm (**A**) The log-rank test *P*-value for Kaplan–Meier survival analysis. (**B**) The clustering heat map visualizing the degree of partitioning in the sample clusters. (**C**) The average silhouette width representing the coherence of clusters.

Molecular classification of PCa was conducted through unsupervised consensus clustering in all tumor samples using the ‘CancerSubtypes’ package of the R software. The optimal number of clusters was determined by the K value. A three-cluster solution (K = 3) with no significant increase in area under the Cumulative Distribution Function (CDF) curve was selected (Figure 6A–C). This was done after evaluating relative changes in the area under the CDF curve and the consensus heat map. The results revealed that 88 patients (38%) in the PCa cohort belonged to Cluster I, 92 (48%) to Cluster II and 133 (64%) to Cluster III. Additionally, the consensus matrix heat map demonstrated that all the three clusters clearly appeared in the individualized clusters in Figure 7A–C. Patients classified as Cluster III had a better prognosis compared with both Clusters I and II (*P*<0.001, log-rank test).

### DEGs and immune cell patterns of Cluster I, Cluster II and Cluster III subtypes

**Figure 8:**
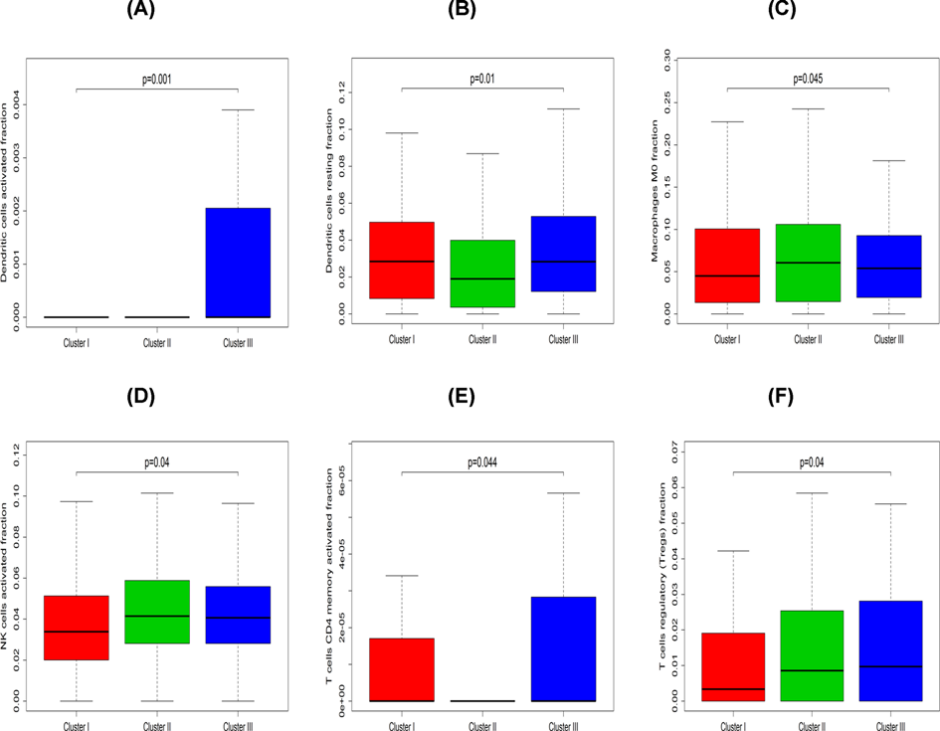
The box plot depicts the association between immune cell and three PCa clusters (**A**–**F**) Represent activated dendritic cells, resting dendritic cells, M0 macrophages, activated NK cells, activated memory CD4 T cells and Tregs, respectively. Kruskal–Wallis test was employed to test the difference among three groups, a *P*-value of less than 0.05 was considered statistically significant.

The molecular subtypes of PCa caused differences in clusters I, II, and III as shown by the activation of specific signaling pathways and different prognoses. The Kruskal–Wallis test was performed to identify subtype-specific genes/LM22 immune cells in each subtype. The results showed that, Clusters I and III were characterized by high levels of resting dendritic cells compared with Cluster II. In addition, Cluster II was enriched by M0 macrophages and activated NK cells. On the other hand, Cluster III was defined by high levels of activated and resting dendritic, activated memory CD4 T cells and Tregs (Figure 8A–F).

**Figure 9:**
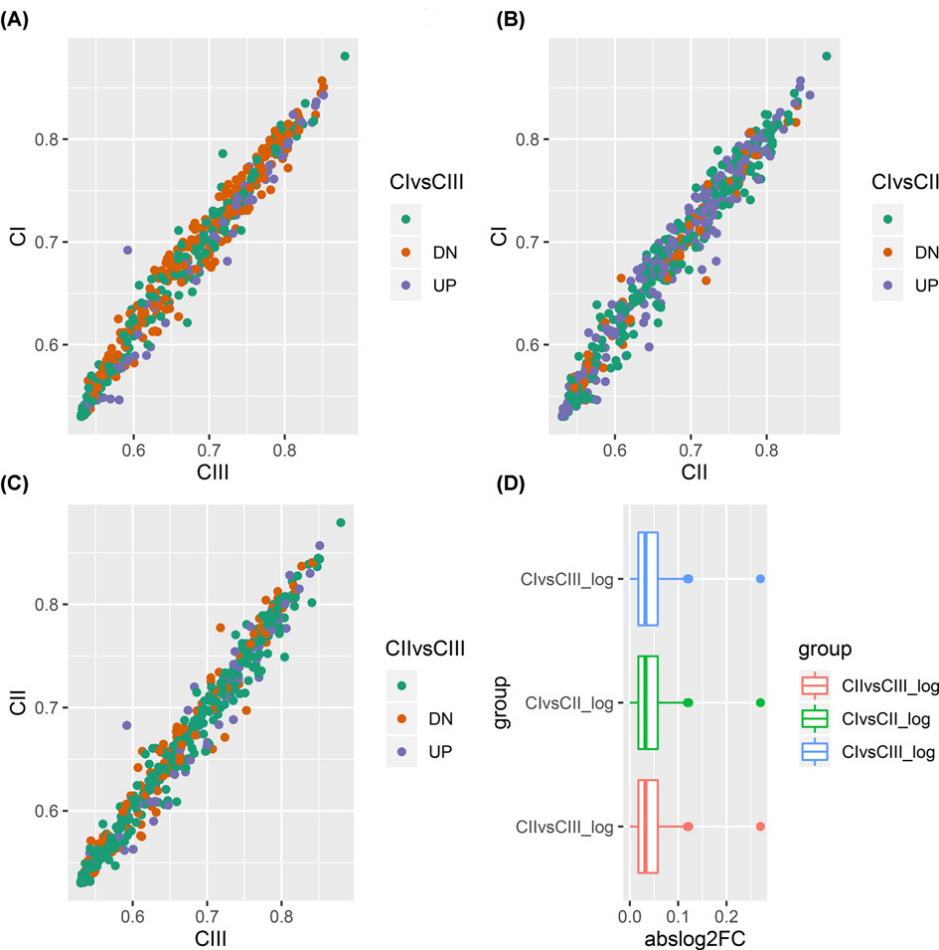
Analysis of differential gene expression of immune-related genes (**A**–**C**) For CI vs CIII, CI vs CIII and CI vs CIII, respectively. Each dot plot shows all immune-related genes. Red represents those whose expression was up-regulated while blue represents those whose expression was down-regulated. (**D**) Bottom right panel: a boxplot showing the distribution of absolute fold changes in the three comparisons made.

The study also sought to uncover differences among PCa molecular subtypes and identify subtype-specific biomarkers. Therefore, the unpaired Student’s *t* test was used to identify DEGs that were significantly associated with each subtype. Analysis of DEGs was conducted with the cut-off point of FDR < 0.05. The results showed that a total of 294 immune-related mRNAs (227 up-regulated genes and 67 down-regulated genes) were differentially expressed in subgroup I. In Cluster II, a total of 292 DEGs (76 up-regulated genes and 216 down-regulated genes) were identified. Finally, a total of 162 DEGs (162 up-regulated genes and 95 down-regulated genes) were observed in subgroup III (Figure 9A–D).

**Figure 10:**
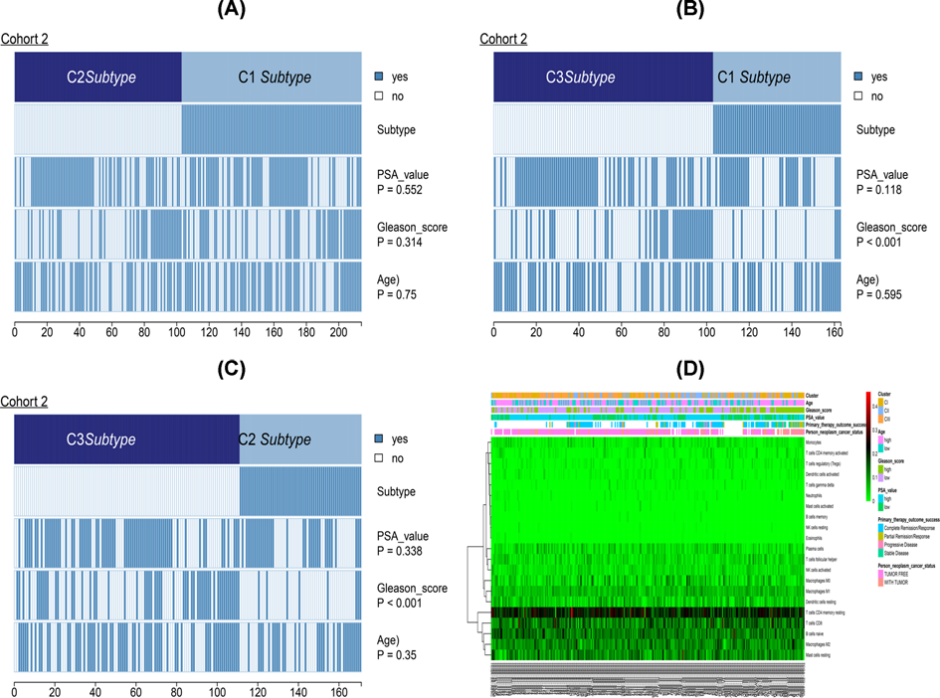
A comparison of PSA values, Gleason score and age among PCa subsets Statistical significance was obtained from the Chi-square test. (**A–C**) Represent CI vs CII, CI vs CIII, CII vs CIII, respectively. (**D**) The heat map illustrates the association between different clinical characters and PCa subsets.

With regard to clinical features among the three clusters, Cluster I had a higher Gleason score and PSA level compared with Clusters II and III. However, there was no significant difference in PSA value and age among the three Clusters (Figure 10A–C). The heat map in Figure 10D illustrates the association of different clinical features among the three subgroups. Statistical significance was obtained from the Kruskal–Wallis test.

### Identification of DEGs and enriched GO and pathway in the subtypes

Functional enrichment analysis for DEGs in CI vs CII, III, CII vs CI, III and C III vs CI, II was performed. A total of 517 GO terms of biological process, 25 GO terms of cellular component and 39 GO terms of molecular function were identified in CI vs CII, III, with a cut-off *P*-value <0.05. Additionally, 476 GO terms of biological process, 25 GO terms of cellular component and 45 GO terms of molecular function were identified in subgroup II compared with the others. Finally, a total of 425 GO terms of biological process, 13 GO terms of cellular component and 18 GO terms of molecular function were identified in subgroup III compared with the other groups.

**Figure 11:**
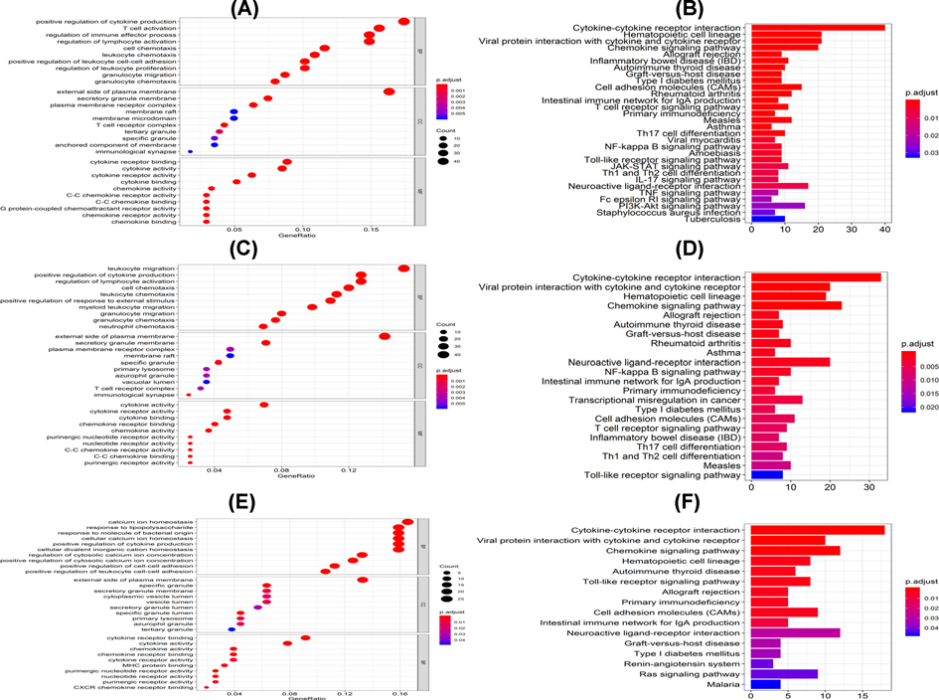
The GO and KEGG analyses of the three PCa clusters (**A,B**) Represent cluster I vs Cluster II. (**C,D**) Stand for cluster I vs cluster III, while (**E,F**) indicate cluster II vs Cluster III.

**Figure 12:**
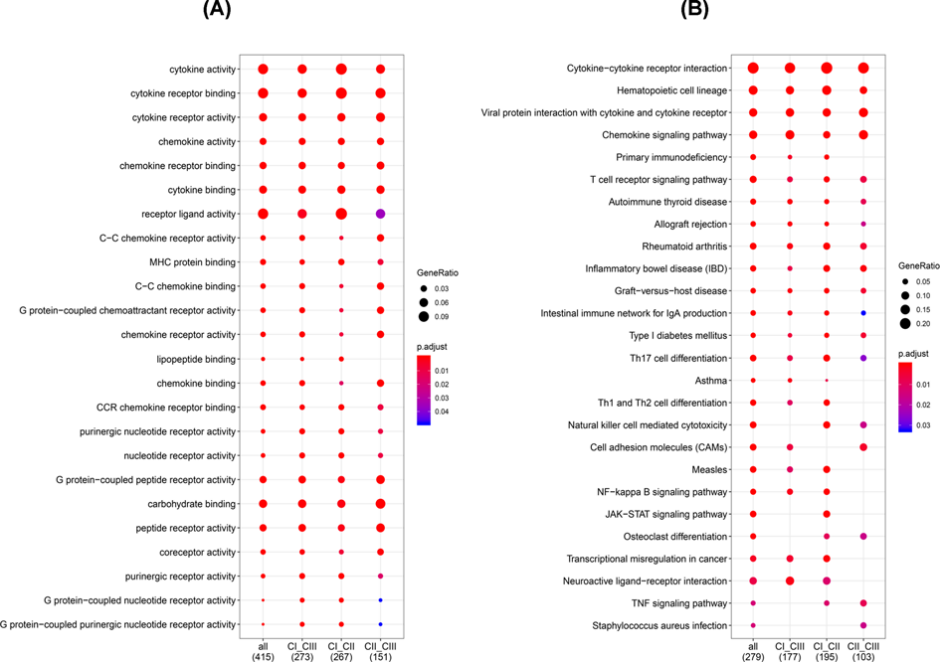
KEGG analysis reveal specific pathways among three groups A comparison of functionally enriched pathways among the three PCa subsets, including (**A**) GO and (**B**) KEGG analyses.

The top GO terms included cytokine activity, immune/inflammatory response and chemokine activity (Figure 11A,C,E). All Clusters were enriched in cytokine activity, chemokine activity and carbohydrate binding. Moreover, Clusters I and II were associated with lipopeptide binding. However, Cluster III was related to receptor ligand activity, G protein-coupled purinergic nucleotide receptor activity and G protein-coupled nucleotide receptor activity (Figure 12A).

Furthermore, all the pathways generated from KEGG analysis were related to immune responses (Figure 11B,D,F). Cluster I was associated with primary immunodeficiency, Th1 and Th2 cell differentiation and the NF-κB signaling pathway. On the other hand, Cluster II was associated with primary immunodeficiency, the JAK−STAT signaling pathway and NK cell-mediated cytotoxicity. Finally, Cluster III was related to Th17 cell differentiation, Cell Adhesion Molecules (CAMs) and the TNF signaling pathway (Figure 12B).

**Figure 13:**
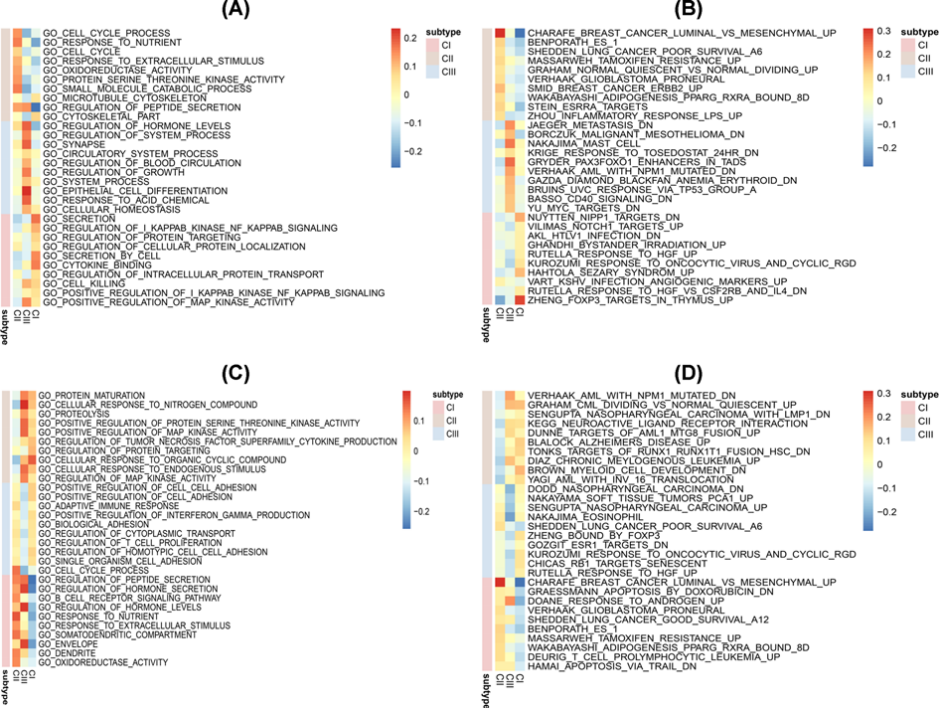
GSEA revealed distinctively enriched gene sets among the three PCa subtypes The up-regulated (**A**) GO and (**B**) KEGG categories as well as the down-regulated (**C**) GO and (**D**) KEGG terms.

GSEA was then performed to identify gene sets enriched in each subtype. The results revealed distinctively enriched gene sets between the subtypes. The number of enriched pathways progressively increased from Cluster I through Cluster III. The study subsequently selected representative gene sets from CI–CIII to build a pathway heat map, which revealed distinct gene sets enriched in each subtype. Cluster I was associated with SMID_BREAST_CANCER_ERBB2 and SHEDDEN_LUNG_CANCER_POOR_SURVIVAL. On the other hand, Cluster II was linked to RUTELLA_RESPONSE_TO_HGF_VS_CSF2RB_AND_IL4_DN and VILIMAS_NOTCH1_TARGETS. Finally, Cluster III was related to BASSO CD40 SIGNALING and VERHAAK AML WITH NPM1 MUTATED (Figure 13A–D). All these similarities corroborated with the molecular and clinical characteristics of the three subtypes identified in the study. This confirmed that the features identified on the three subtypes were correct.

### Subclass-associated gene mutations

**Figure 14:**
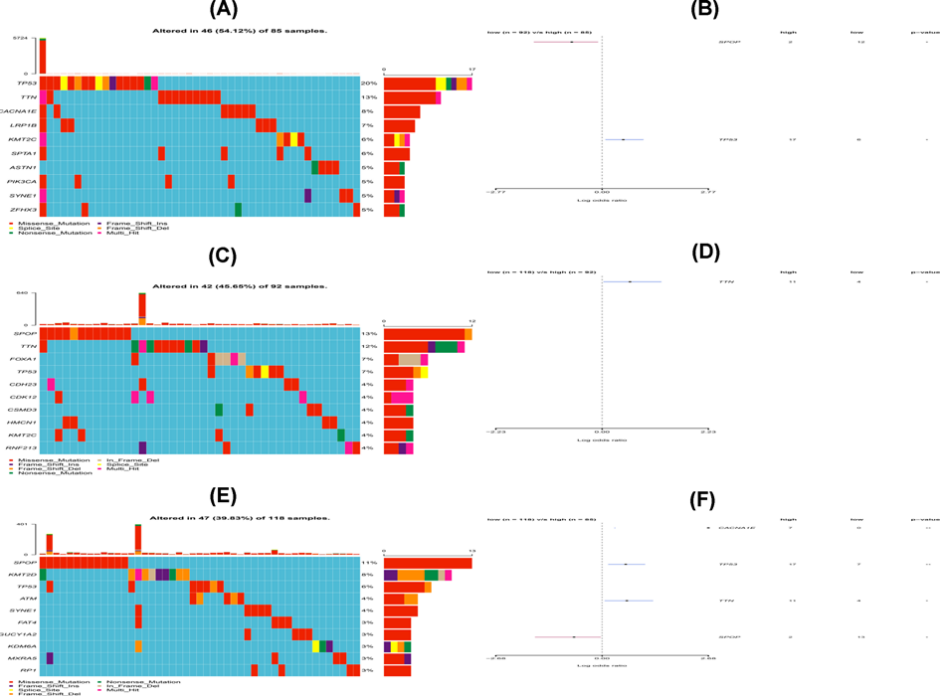
Mutation analysis of the three PCa subsets (**A,C,E**) Gene mutation profiles of the highly mutated genes in the three subtypes. (**B,D,F**) The forest plots show the results of comparisons in gene mutations in CI, CII and CIII (*P, 0.05, **P, 0.01, ns: not significant).

The association between the three subtypes and number of somatic mutations was also explored. The mutation profiles of the highly mutated genes are shown in Figure 14A–F. Notably, SPOP displayed a higher rate of mutation in Cluster I while TP53 exhibited a higher mutation rate in Cluster II. In Cluster III, however, CACNA1E, TTN and TP53 exhibited higher mutation rates.

### Sensitivity to immuno/chemotherapies in Cluster I, II and III subtypes

**Figure 15:**
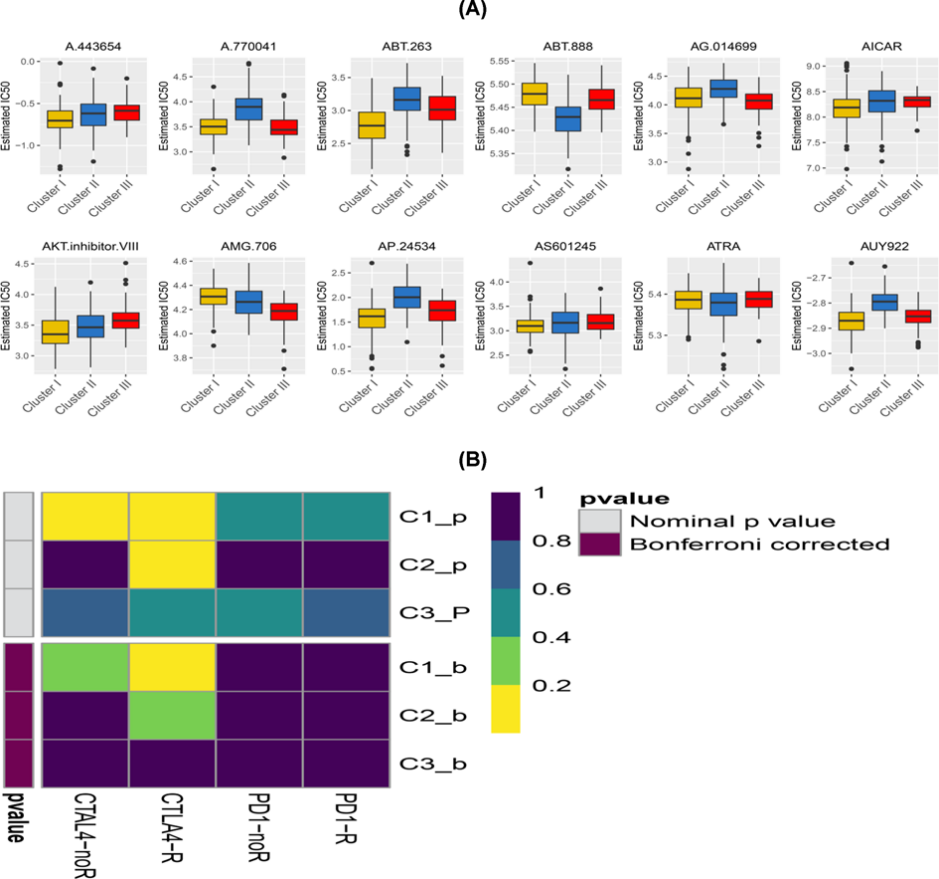
Differential putative chemotherapeutic and immunotherapeutic responses The box plots of the estimated IC_50_ values for chemotherapeutic drugs are shown in (**A**) for the CI, CII and CIII PCa subsets. (**B**) Submap analysis revealed that Cluster I could be more sensitive to immunotherapy (Bonferroni-corrected *P*<0.05).

Although immunological checkpoint drugs have not been approved for use as the conventional treatment for PCa, the TIDE algorithm was used to predict the likelihood of responding to immunotherapy. The findings showed that Cluster I was more likely to respond better to immunotherapy compared with Clusters II and III (*P*<0.05). In addition, TIDE was predicted through subclass mapping method that compared the expression profiles of the three PCa subtypes with a published dataset containing 47 melanoma patients who responded to immunotherapy. Treatment with CTLA4 showed more promising results in Cluster I as shown in Figure 15A (Bonferroni correction *P*<0.05).

Given that chemotherapy is currently commonly used against PCa, the study sought to evaluate response by the three subtypes to the frequently used drugs. Therefore, the prediction model was trained on the GDSC cell line dataset by ridge regression and a satisfactory prediction accuracy of ten-fold cross-validation was evaluated. The IC_50_ value for each sample was estimated in the TCGA dataset based on the predictive model of the two chemo drugs. Ultimately, a total of 12 targeted inhibitors were identified as potential drugs against PCa in the three subtypes (Supplementary Table S3). Notably, it was observed that all the clusters had significant differences in the estimated IC_50_ values although Cluster I may have been more sensitive to commonly used chemotherapy as shown in Figure 15B (AKT.inhibitor.VIII *P*<0.001, ATRA *P*≤0.001).

## Discussion

PCa is a major disease that threatens human health and is the most common malignancy in Western countries [26]. Over time, cancer has been recognized as an adaptive and complex system and achieving effective treatment using single-target drugs has become increasingly difficult. Immunotherapy is considered a promising alternative to cancer treatment as it may help overcome the challenge of drug resistance. Presently, satisfactory results have been observed in the use of immunotherapy against certain cancers including malignant melanoma. However, immunotherapy has not been shown to be effective in the treatment of PCa [27,28].

TILs may be used as effective targets for drugs to improve clinical outcomes. In addition, with the increasing focus on immune checkpoint therapy, distribution of TIIC has spurred great research interest. Previous studies showed that the degree of TIIC could mainly be obtained through Immunohistochemistry (IHC). However, IHC markers are not precise and multiple TILs may express the same markers on the cell membrane. This leads to the inaccurate measurement of TIIC density in PCa tumor tissues [29]. Therefore, this study aimed to predict immune cell infiltration using the CIBERSOFT algorithm in the TCGA PCa cohort. Additionally, therapeutic efficacy of immunotherapy in different subtypes was evaluated in order to find a specific subtype that responded well to immunotherapy.

In the current study, the CIBERSOFT algorithm was used to assess the infiltration of immune cells in normal tissues as well as PCa tumor and adjacent tumor tissues. The results revealed considerable differences in immune cell components within and between groups. This research also revealed details on the subpopulations of LM22 immune cells in PCa. Macrophages and NK cells were previously detected in PCa [30,31]. In addition, NK cells and macrophages can be used in androgen deprivation therapy. Moreover, a large number of NK cells can reduce the risk of tumor progression although a high proportion of macrophages is able to increase the risk of biochemical recurrence [32]. It is therefore urgent that new and highly effective prognostic markers are added to the currently available ones in order to improve precision.

The present study also uncovered in detail, the infiltration of LM22 immune cell subsets in PCa. The proportion of infiltrated T cells was more than 39%. Out of this, resting memory CD4 T cells accounted for 20%, CD8 T cells took up 13%, helper follicular T cells accounted for 3% while the proportion of activated memory CD4 T cells, Tregs and T cells γδ was less than 1%. In addition, total macrophages had the second highest proportion of 13% (Table 1). Moreover there was a significant difference (*P*<0.05) in the proportions of total T cells, B cells, activated dendritic cells, M0 and M1 macrophages, activated NK cells and memory CD4 T cells between the normal and adjacent tumor tissues. Furthermore, resting dendritic cells and neutrophils increased in the adjacent tumor tissues.

In contrast, memory B cells, M2 macrophages, activated and resting mast cells, activated and resting NK cells, plasma cells and follicular helper T cells were mainly enriched in the normal tissues. However, the mechanisms behind the activation of NK cells, macrophages, activated dendritic cells, deactivation of B cells and plasma cells in PCa remain unclear. Macrophages and NK cells were previously detected in PCa [33,34]. Additionally, Philippe et al. reported that patients under androgen-deprivation therapy could use NK cells and macrophages. Moreover, a large number of NK cells could reduce the risk of tumor progression although a high proportion of macrophages could increase the risk of biochemical recurrence [32].

The prognostic importance of immune cell infiltration has been determined in various solid tumor types [35–37]. In this study, univariate and multivariate Cox analyses showed that only memory B cells were significantly correlated with RFS. In addition, patients with a higher density of memory B cells had a shorter RFS time. The KM curve of memory B cells showed a similar trend. Several studies reported that tumor-infiltrating B cells were a positive prognostic factor in breast cancer [38], ovarian cancer [39], non-small cell lung cancer [40] and other cancers [41,42]. In addition, Spear et al. demonstrated that B cells are actively involved in the tumor microenvironment and can produce immune-stimulating factors that may support adaptive anti-tumor immune responses in pancreatic ductal adenocarcinoma [43].

PCa can be reliably divided into three subtypes using the NMF method. Based on the results, the three subtypes were significantly associated with patient survival. Patients grouped as Cluster III had a better prognosis compared with Cluster II and Cluster I. Moreover, Cluster I was defined by high levels of macrophage infiltration and Cluster II was associated with B- and T-cell infiltration. However, Cluster III was defined by high levels of mast cell, neutrophil and NK cell infiltration. Each cluster had its own feature-rich terminology compared with each DEG cluster. Therefore, these three subtypes differed in both RFS and molecular characteristics. Furthermore, submap and TIDE analyses suggested that treatment with CTLA4 was more promising in Cluster I. The GDSC database, also implied that Cluster I could be more sensitive to commonly used chemotherapies compared with Clusters II and III. Therefore, it is possible that Cluster I may benefit more from the combination of chemotherapy and immunotherapy. Additionally, Cluster I may be more sensitive to the commonly used chemotherapies (AKT.inhibitor.VIII *P*<0.001, ATRA *P*≤0.001).

## Conclusions

Analysis of the LM22 subsets using the CIBERSORT deconvolution algorithm provided comprehensive information on the pattern of immune cells in PCa. The findings also revealed an important role in predicting clinical outcomes. This comprehensive assessment of the LM22 immune cell infiltration model in TME provides insights on how tumors respond to immunotherapy and may help clinicians explore the development of new drugs. As our study results were derived from bioinformatics analysis, further clinical studies are needed to confirm these results.

## Supplementary Material

Supplementary Tables S1-S3

## Data Availability

Data could be obtained from TCGA website.

## Abbreviations

CDF: cumulative distribution function
CTLA4: Cytotoxic T-Lymphocyte Associated Protein 4
DEG: differentially expressed gene
FPKM: Fragments per Kilobase of exons per million mapped reads
GDSC: Genomics of Drug Sensitivity in Cancer
GO: gene ontology
GSEA: Gene Set Enrichment Analysis
GTEx: the Genotype-Tissue Expression
IC_50_: half-maximal inhibitory concentration
IHC: immunohistochemistry
KEGG: Kyoto Encyclopedia of Genes and Genomes
LM22: leukocyte signature matrix subset
NK: natural killer
NMF: Nonnegative Matrix Factorization
PCa: prostate cancer
PFS: progression-free survival
PSA: Prostate-Specific Antigen
RFS: Recurrence-Free Survival
TCGA: The Cancer Genome Atlas
TIDE: Tumor Immune Dysfunction and Exclusion
TIICs: Tumor infiltrating immune cells
TIL: Tumor Infiltrating Leukocyte
Treg: regulatory T cell
