# Supplementary material for: Identification of immune-based prostate cancer subtypes using mRNA expression
Source: Biosci Rep. 2021 Jan 4;41(1):BSR20201533. doi: 10.1042/BSR20201533 (PMC7785043; doi:10.1042/BSR20201533)
Supplement: Supplementary Tables S1-S3 [file BSR-2020-1533_supp.pdf]

Supplementary Table 1: the 22 human leukocyte signature matrix(LM22) immune cells.

LM22
B cells naive
B cells memory
Plasma cells
T cells CD8
T cells CD4 naive
T cells CD4 memory resting
T cells CD4 memory activated
T cells follicular helper
T cells regulatory (Tregs)
T cells gamma delta
NK cells resting
NK cells activated
Monocytes
Macrophages M0
Macrophages M1
Macrophages M2
Dendritic cells resting
Dendritic cells activated
Mast cells resting
Mast cells activated
Eosinophils
Neutrophils

Supplementary Table 2: A total of 138 drugs showed potential for the treatment of cancer.

Drugs
A.443654
A.770041
ABT.263
ABT.888
AG.014699
AICAR
AKT.inhibitor.VIII
AMG.706
AP.24534
AS601245
ATRA
AUY922
Axitinib
AZ628
AZD.0530
AZD.2281
AZD6244
AZD6482
AZD7762
AZD8055
BAY.61.3606
Bexarotene
BI.2536
BIBW2992
Bicalutamide
BI.D1870
BIRB.0796
Bleomycin
BMS.509744
BMS.536924
BMS.708163
BMS.754807
Bortezomib
Bosutinib
Bryostatin.1
BX.795
Camptothecin
CCT007093
CCT018159
CEP.701
CGP.082996
CGP.60474
CHIR.99021

CI.1040
Cisplatin
CMK
Cyclopamine
Cytarabine
Dasatinib
DMOG
Docetaxel
Doxorubicin
EHT.1864
Elesclomol
Embelin
Epothilone.B
Erlotinib
Etoposide
FH535
FTI.277
GDC.0449
GDC0941
Gefitinib
Gemcitabine
GNF.2
GSK269962A
GSK.650394
GW.441756
GW843682X
Imatinib
IPA.3
JNJ.26854165
JNK.9L
JNK.Inhibitor.VIII
JW.7.52.1
KIN001.135
KU.55933
Lapatinib
Lenalidomide
LFM.A13
Metformin
Methotrexate
MG.132
Midostaurin
Mitomycin.C
MK.2206
MS.275
Nilotinib
NSC.87877
NU.7441
Nutlin.3a

NVP.BEZ235
NVP.TAE684
Obatoclax.Mesylate
OSI.906
PAC.1
Paclitaxel
Parthenolide
Pazopanib
PD.0325901
PD.0332991
PD.173074
PF.02341066
PF.4708671
PF.562271
PHA.665752
PLX4720
Pyrimethamine
QS11
Rapamycin
RDEA119
RO.3306
Roscovitine
Salubrinal
SB.216763
SB590885
Shikonin
SL.0101.1
Sorafenib
S.Trityl.L.cysteine
Sunitinib
Temsirolimus
Thapsigargin
Tipifarnib
TW.37
Vinblastine
Vinorelbine
Vorinostat
VX.680
VX.702
WH.4.023
WO2009093972
WZ.1.84
X17.AAG
X681640
XMD8.85
Z.LLNle.CHO
ZM.447439

Supplementary Table 3: Atotal of 12 targeted inhibitors were identified as potential drugs against PCa in the three subtypes.

Drugs	P value
A.443654	0.003
A.770041	0.004
ABT.263	0.023
ABT.888	0.014
AG.014699	0.023
AICAR	0.036
AKT.inhibitor.VIII	0.002
AMG.706	0.005
AP.24534	0.007
AS601245	0.043
ATRA	0.028
AUY922	0.049
